# The Use of Imatinib Mesylate as a Lifesaving Treatment of Chronic Myeloid Leukemia Relapse after Bone Marrow Transplantation

**DOI:** 10.1155/2009/357093

**Published:** 2009-10-22

**Authors:** Monika Conchon, Sabri S. Sanabani, Israel Bendit, Carla Luana Dinardo, Lucia Dias, Dalton de Alencar Fischer Chamone, Pedro Enrique Dorlhiac-Llacer, Frederico Luiz Dulley

**Affiliations:** ^1^Disciplina de Hematologia, da Faculdade de Medicina, da Universidade de São Paulo, 05403-000 São Paulo, Brazil; ^2^Laboratorio de Cytogenetica, da Disciplina de Hematologia, da Faculdade de Medicina, da Universidade de São Paulo, 05403-000 São Paulo, Brazil; ^3^Fundação Pro-Sangue/ Hemocentro de São Paulo, 05403-000 São Paulo, Brazil

## Abstract

We describe the response of imatinib as lifesaving treatment of chronic myeloid leukemia (CML) relapse in seven patients who underwent allogeneic bone marrow transplantation (alloBMT) at our institution over a period of 4 years. Retrospective analysis of their medical records revealed that a mean age at transplant was 45.2 years. The median time to diagnosis was 7.4 years after transplant. At relapse, four, two, and one patients were classified as having hematologic, major molecular, and cytogenetic relapse, respectively. At imatinib initiation, five had CML in a chronic phase, while one patient was diagnosed as having accelerated phase and blast crisis. All these patients could be evaluated for the therapeutic efficacy. At a mean of follow-up of 1.9 years of therapy, all evaluable patients achieved major molecular response without compromising safety. Consistent with available data, our results indicate that imatinib is safe and effective treatment option for patients with relapse after BMT.

## 1. Introduction

Chronic myeloid leukemia (CML) is a myeloproliferative disease that represents 15%–25% of all leukemias. The disease was the result of a reciprocal translocation between chromosome 22 and chromosome 9, the so-called Philadelphia translocation [1]. This rearrangement joins the *c-ABL* gene on chromosome 9 and *BCR* on chromosome 22 creating a *BCR-ABL* fusion gene, which codes for a 190–230 kDa, *BCR-ABL* fusion protein with elevated tyrosine kinase activity. The upregulated *BCR-ABL* fusion protein includes the tyrosine kinase domain, which likely contributes to dysregulation of the mechanism of cellular signals transduction, normally involved in controlling apoptosis, proliferations, and cell-cell adhesions, and thus promoting leukemogenesis [2]. The abrogation of *BCR-ABL* function has become a model for development of targeted therapies. The enzymatic inhibition of *BCR-ABL* using Imatinib mesylate (GleevecTM, STI571; Novartis Pharmaceutical Corp., East Hanover, NJ) has been shown to possess potent in vitro and in vivo activities in preclinical studies [3, 4]. The activity of this inhibitor in patients with previously treated CML was further confirmed in early clinical trials [5–7]. Recently, the landmark International Randomized Study of Interferon versus STI571 (IRIS) study has shown cytogenetic responses in 87% of their patients after 5 years of imatinib as a primary treatment [8]. Over the past few decades, allogeneic bone marrow transplantation (allo-BMT) has been used to treat CML and other malignant and nonmalignant diseases. This therapeutic option brought a fair amount of success and proved to prolong survival. However, this success is frequently not maintained since leukemic relapse substantially occurs in 30% to 60% of patients who undergo transplantation during the advanced stages of disease (accelerated phase and blast crisis) and 5% to 20% of those who undergo transplantation during the chronic phase [7]. Moreover, the impact of this potential treatment is limited because it depends considerably on the patient's age and availability of a genetically cross matching donor with the same tissue type to prevent rejection [9]. The infusion of donor lymphocytes (DLI) into CML patients who have relapsed following an allo-BMT has been frequently used and proved to effectively treat 70%–80% of patients with relapsed chronic leukemia. However, this therapeutic option is less effective in patients with more advanced disease and may also be associated with severe marrow aplasia, high incidence of chronic graft-versus-host disease (GVHD), and transplant-related mortality [10–12]. Several studies have shown that the administration of imatinib is safe and highly effective in controlling leukemic relapses after allo-BMT [13, 14]. These compelling facts have made us adopt imatinib therapy as the preferred treatment at our institution. In this study, we performed a retrospective analysis to determine the efficacy of imatinib administration in treating leukemic relapses after allo-BMT.

## 2. Materials and Methods

From January 2005 to January 2009, a total of 7 cases with primary relapse of CML after allo-BMT undergoing treatment with imatinib were found after a retrospective search of the CML database at the Department of CML Ambulatory of the Hospital das Clinicas, São Paulo University. Patients' medical records were reviewed for the following: sex, age, date of CML diagnosis, time from diagnosis to transplant, time from transplant to relapse, disease stage at relapse, and other variables as shown in Table 1. The extent of CML was staged (chronic, accelerated or blast phase) according to the 2008 revision of the World Health Organization staging system. In all patients, complete blood counts were performed weekly for the first month of follow-up and monthly thereafter. Quantification of *BCR-ABL* transcript numbers was measured every 3 months by real-time PCR and conventional bone marrow cytogenetics were performed every 6 months and then every 12 months.

Response criteria were as previously described [15, 16]. Cytogenetic response was classified as complete (no Ph chromosome-positive cells in metaphase in the bone marrow), partial (1%–34% Ph chromosome-positive cells), or minor (35%–90% Ph chromosome-positive cells). Major cytogenetic responses included complete and partial cytogenetic responses [17]. Molecular response was considered major if the *BCR-ABL* level was below 0.1% and complete when it was negative.

Toxicities were assessed according to the National Cancer Institute Common Toxicity Criteria version 3.0 (National Cancer Institute, Bethesda, MD). Posttransplant allogeneic hematopoietic chimerism was evaluated by fluorescent-based PCR amplification and capillary gel electrophoresis of microsatellite DNA STR markers in sequential peripheral blood and bone marrow samples from the recipients using established techniques [18].

Curves of overall survival and progression free survival were estimated using the Kaplan and Meier method.

## 3. Results

A total of seven patients were treated with imatinib after having undergone alloBMT. The subjects' mean age at transplant was 45.2 years (Table 1). Males contributed to 57.1% of the sample. All 7 patients received an allogeneic peripheral blood progenitor cell from HLA-identical sibling. The median time to diagnosis was 7.4 years after transplant. At the time of relapse, four patients were classified as having a hematologic relapse, two as having a major molecular relapse, and one as having a cytogenetic relapse. The mean time from alloBMT to detection of relapse was 4.02 years. Two patients received a DLI as a salvage therapy after relapse to enhance donor engraftment without response. Of the seven patients, five had CML in a chronic phase at the time of imatinib initiation, while one patient was diagnosed as having accelerated phase and blast crisis. Imatinib was given at a dose of 400 mg/d (five patients) or 600 mg/d (two patients). One patient failed to show any response to treatment. This latter patient was in blastic crisis before imatinib administration and died of rapid disease progression 7 months after initiation of therapy, before hematologic recovery. Eventually, all patients could be evaluated for the therapeutic efficacy. At a mean of follow-up of 1.8 years of imatinib therapy, all but one patient had achieved complete hematologic, cytogenetic, and major molecular response. The mean time of major molecular response was 5.6 months. Response to imatinib was then evaluated based on relapse classification and found that all but one patient with hematologic relapse achieved major molecular response. Analyses of the safety data in patients who remained alive indicate that the treatment was generally well tolerated and that most of toxicities were mild and transient. Four patients experienced grade 2 hematological toxicities, while one patient developed grade 2 alterations in hepatic enzymes. Complete donor chimerism was achieved after transplantation in all six evaluable patients. The estimated overall and progression-free survivals were 7.1 and 2.1 years, respectively.

## 4. Discussion

A number of therapeutic interventions have met with varying degrees of success in the treatment of CML patients with relapse after allo-BMT. These options include a second transplant [19, 20], conventional cytotoxic chemotherapy, and interferon-alpha [21]. Each treatment, however, has limitations. For example, although the option of second allo-BMT offers a prospect of cure, it is limited to young patients with good performance status and is associated with a very poor outcome [10, 21, 22]. Evidence from several reports indicated a high remission rate (70%–80%) after DLI in chronic phase CML. However, the success of this option has been limited by short duration of response, myelosuppression, significant GVHD, and rare long-term survival [1, 5, 19]. It is therefore important to consider the effects of the alternative therapies listed above on outcomes. 

The recent use of imatinib mesylate as new therapeutic approach is eventually become accepted as the preferred treatment of posttransplant relapse setting and offers a number of potential advantages over DLI including fewer adverse effects and relatively rapid and durable response. Apart from our study, several anecdotal and clinical trials have examined the use of imatinib in patients with relapse after BMT. The largest of these was a prospective phase II open label multicenter study by Hess et al. [13] who examined the use of imatinib therapy in 44 relapsed patients. In this 494-day median follow-up study, patients were treated with imatinib at starting dose of 400 mg/d and escalated to 600 mg/d to 800 mg/d if patients did not achieve MMR. Of the 37 patients assessable for efficacy analysis, a total of 23 patients (62%) had a CMR during the initial 9 months, which improved to 70% during follow-up. Therapy was generally well tolerated, with a main adverse event of neutropenia/leucopenia. The review by Palandri and coworkers [23] of the outcomes of 16 patients who received imatinib as first or second line therapy showed that 93% of this group achieved a molecular negativity after 3–27 months and 75% had negative reverse transcriptase-polymerase chain reaction after 12–45 months. Similarly, Hayat et al. [24] found, in a group of 14 patients who relapsed postallo-BMT from the preimatinib era, that 93% had achieved excellent response to imatinib with a median time of 4 months.

In this study, although the sample size is small, imatinib maintained MMR in all 6 evaluable patients for an average time of 21 months of follow-up. All patients are still on treatment with imatinib and have full-donor chimerism according to the last analysis. Generally, the results presented here compare favorably with the outcomes from previous studies [13, 19, 25] and lend support to the conclusion that the imatinib therapy is efficiently improving outcome even further in patients with relapse after BMT particularly for chronic phase CML without compromising safety.

Although the introduction of imatinib has obviously been a major step forward a high objective response and disease stabilization rate in patients with relapse after BMT, several questions still remain open. Among them, how well does imatinib perform over the long-term of therapy? Is there a tendency for some patients to gradually relapse overtime passage? Can patients eventually stop their therapy without relapsing? To provide answers to these questions, additional double-blind imatinib-placebo controlled research of long-term follow-up is needed to further evaluate the safety of a long-term, continuous therapy with the imatinib in a large number of patients with relapse after BMT.

## Figures and Tables

**Table 1:** Patients characteristics, CML stage and IM response. F: Female, M: Male, Δsis: Diagnosis, CP: chronic phase, BC: blastic crisis, AP: accelerated phase, IM: imatinib, Mol: molecular, Hem: hematologic, Cyt: cytogenetic, Hep: hepatic, MMR: major molecular reponse, NR: no reponse.

Case number	Sex	Age (years)	Δsis	1st line therapy	Type of relapse at IM therapy	IM dose (mg/d)	Maximum IM reponse	Time to acheive MMR (month)	Toxicity	Current RT-PCR status
1	M	28	CML (CP)	IM	Mol	400	MMR	3.7	No	Negative
2	F	44	CML (CP)	IM	Cyt	400	MMR	9.2	No	Positive
3	M	50	CML (CP)	3X	Hem	400	MMR	3	No	Negative
4	F	47	CML (CP)	1X	Hem	400	MMR	6	Hem	Positive
5	M	55	CML (BC)	IM	Hem	600	NR	—	Hem	—
6	F	48	CML (CP)	IM	Mol	400	MMR	2	Hep	Positive
7	F	45	CML (AP)	IM	Hem	600	MMR	9.5	Hem	Positive
